# Inflammation, Peripheral Signals and Redox Homeostasis in Athletes Who Practice Different Sports

**DOI:** 10.3390/antiox9111065

**Published:** 2020-10-30

**Authors:** Simone Luti, Alessandra Modesti, Pietro A. Modesti

**Affiliations:** 1 Department of Biomedical, Experimental and Clinical Sciences “Mario Serio”, University of Florence, 50134 Florence, Italy; simone.luti@unifi.it; 2 Department of Experimental and Clinical Medicine, University of Florence, 50134 Florence, Italy; pamodesti@unifi.it

**Keywords:** cytokines, redox homeostasis, sport performance

## Abstract

The importance of training in regulating body mass and performance is well known. Physical training induces metabolic changes in the organism, leading to the activation of adaptive mechanisms aimed at establishing a new dynamic equilibrium. However, exercise can have both positive and negative effects on inflammatory and redox statuses. In recent years, attention has focused on the regulation of energy homeostasis and most studies have reported the involvement of peripheral signals in influencing energy and even inflammatory homeostasis due to overtraining syndrome. Among these, leptin, adiponectin, ghrelin, interleukin-6 (IL6), interleukin-1β (IL1β) and tumour necrosis factor a (TNFa) were reported to influence energy and even inflammatory homeostasis. However, most studies were performed on sedentary individuals undergoing an aerobic training program. Therefore, the purpose of this review was to focus on high-performance exercise studies performed in athletes to correlate peripheral mediators and key inflammation markers with physiological and pathological conditions in different sports such as basketball, soccer, swimming and cycling.

## 1. Introduction

In elite athletes, there is large disparity among the training protocols; the effects on oxidative stress (OS) and inflammatory cytokines are still not well known. Is important to highlight that excessive training loads are able to trigger the syndrome known as overtraining syndrome (OTS), a phenomenon in which there is an increase in pro-inflammatory markers and consequently a decrease in sport performance [1]. Therefore, the correct prescription of training is essential. In a previous review, we concluded that chronic training seems to be responsible for some perturbations in different metabolic hormones linked to inflammation and in oxidative stress [1]. In this review, we introduce the importance of summarizing the current state of understanding high-performance exercise and its associations with inflammatory mediators and oxidative stress. The intention is to identify and evaluate the most relevant studies in this area and to identify future important investigations. We divide the manuscript into the following: (a) description of the sports considered and the reports of related peripheral signs of inflammation, trying to discern literature data into studies on resting state, moderate and extensive exercise, and (b) oxidative stress and redox homeostasis related to the same specific sports. In the conclusions, we summarize and explain the importance of discovering potential biomarkers that could be used to prevent conditions like overtraining. We believe that the topic of this review article is interesting because there is a need to correlate peripheral levels of specific mediators with physiological/pathological conditions in sports performers. We searched in PubMed using terms related to inflammation and adipocytokine and to the four specific sports considered: cycling, basketball, soccer and swimming. We chose these sports for their characteristics. Soccer and basketball require specific skills such as the ability to move at high speed or change direction quickly. As a result, soccer and basketball athletes tend to possess high strength, power and agility. The metabolism used in these sports is predominantly alternate anaerobic/aerobic; in particular, in basketball, the aerobic demand is less than soccer, but more than baseball and volleyball. Swimming and cycling are effective, low-impact forms of cardio exercise and are both forms of predominantly aerobic exercise, with both being full body workouts. The sports considered can be grouped into two subgroups because of the fiber type profiles in the muscles they require. Swimming and cycling are endurance and long-distance sports characterized mainly by slow twitch (Type I) fibers, while basketball and soccer are associated with fast twitch (Type II) fibers which are mainly involved in quick bursts with great power.

## 2. Inflammation and Peripheral Signals in Sports

Peripheral mediators could be used to monitor both long- and short-term effects in elite athletes during training exercise. Leptin, adiponectin and ghrelin exert important effects on the hormonal regulation in response to acute exercise and chronic training and are related to each other. It is interesting to evaluate the effects of specific training on these molecules and on metabolic state in athletes since cytokine responses are linked to changes in physical performance. Jürimäea et al. [2] found that peripheral signals are important in the regulation of energy homeostasis and they communicate the status of body energy to the hypothalamus that plays a central role in integrating them. Leptin is a signaling hormone that decreases with weight loss and its serum levels are correlated with body mass index (BMI). In skeletal muscle, leptin stimulates fatty acid oxidation, increases glucose uptake and prevents accumulation of lipids in non-adipose tissue [3,4]. Legakis et al. reported that in short-term exercise, the plasma leptin concentration is reduced after exercise [5] and in response to long-term exercise, moreover they reported that plasma leptin showed different variations in competitive endurance rates [6]. In summary, the effects of chronic short- and long-term exercise programs on circulating leptin vary among the different studies [7]. Bouassida et al. [8] reported that in well-trained athletes, leptin concentration is not modified after short-term exercise (less than 60 min) and decreases after long-term exercise (more than 60 min). Olive et al. specify that a bout of exercise with a long duration and at a moderate intensity causes a decrease in plasma leptin concentration for up to 72 h post-exercise. On the contrary a short-duration, maximal-intensity exercise has no effect suggesting that the effect on leptin plasma level can be independent of the energy balance and dependent on the type of exercise practiced [6]. Adiponectin decreases glucose production in the liver, improves the uptake of glucose and fatty acid oxidation in skeletal muscle and modulates metabolic effects operating in an autocrine and endocrine manner. It is involved in increasing insulin sensitivity and its plasma concentration may be modified during physical exercise [1]. Well-trained athletes show high baseline adiponectin in plasma concentration and submaximal exercise alone does not alter adiponectin concentration but it exerts an important role in the hormonal regulation [9]. In highly trained athletes, levels of circulating adiponectin changed after an acute exercise: its concentration decreased immediately after maximal or acute exercise and on the contrary, during the recovery period (30 min), the adiponectin level increased [10]. A decrease in post-exercise adiponectin and leptin values in rowers with a lower performance capacity may be indicative of the inadequate recovery of these athletes [11]. Moreover, chronic exercise in short-term (<12 weeks) training and long-term (>12 weeks) training has opposite effects on leptin and adiponectin levels, suggesting that exercise training protocols that induce a reduced fat mass are accompanied by lower leptin concentrations and higher resting adiponectin plasma levels. As reported by several authors in endurance-trained athletes and in post-exercise, the leptin level is reduced and ghrelin is increased [12]. Ghrelin is inversely associated with body mass index [13]. Studies on the effect of exercise on the ghrelin plasma level show that it is a mediator of GH expression in response to moderate-high-intensity exercise [14]. Jürimäe, J et al. showed, immediately after 30 min of high-intensity exercise, the circulating ghrelin level was 7% higher than before [15]. In a subsequent publication, the same authors found plasma ghrelin significantly increased (15%) when measured 30 min after a prolonged low-intensity single exercise session but not immediately after the exercise [16]. To explain these apposite findings, the authors suggest that in rowers, during prolonged endurance training sessions, there is a negative energy balance and this changes in the ghrelin concentration. Moreover these changes are related to the distance covered. In the first study, they reported that the maximal test performed for about 20 min resulted in a significant post-exercise increase in plasma ghrelin concentrations related to the exercise-induced energy expenditure [15]. Moreover, they reported that the plasma ghrelin response depends on the amount of total work performed [16]. According to several authors, the plasma level of ghrelin is not modified in response to acute aerobic exercise, while acute resistance exercise induces a decline in circulating ghrelin levels [17,18,19]. We can conclude that acute aerobic exercise (running and cycling exercise) does not alter plasma ghrelin levels; on the contrary, acute resistance exercise induces a decrease in plasma ghrelin levels. We suggest that plasma ghrelin concentrations are influenced by metabolic state, energy balance and exercise intensity and duration. Therefore, understanding the roles of these peripheral signals on the complex mechanisms of adaptations to acute exercise and chronic training will allow for using them as indicators of overreaching/overtraining. The increase in cytokines activates the sympathetic nervous system that induces modification in catecholamines, glucocorticoids and gonadal hormones. In a recent review [1], we concluded that chronic training stress is responsible for perturbations in several metabolic hormones related to inflammation. Leptin exerts a strong proinflammatory activity linked to its resemblance to IL-6. On the contrary, ghrelin shows a reduction in inflammation in several non-autoimmune inflammatory diseases. Adiponectin is another signaling hormone showing an anti-inflammatory effect. Well-trained athletes show high plasma adiponectin levels, suggesting that training could modify the adiponectin response and therefore a decrease in its plasma concentration may be a sign of muscle fatigue up to the overreaching/overtraining syndrome. We concluded that some peripheral signals, in particular adiponectin, could play a role in the adaptation to chronic exercise training and inflammation due to a strenuous training. Rowlands et al. [20] hypothesized that the release of tumor necrosis factor-alpha (TNFa) and IL6 after an ultra-endurance run is a consequence of an immune response to localized muscle damage to induce muscle reconstruction after exhaustive exercise. Multi-stage exercise over consecutive days without an adequate recovery period could induce an inflammatory process similar to infection. Prolonged intense physical activity could lead a cascade of metabolic and immune changes in the body that, in professional athletes, could reduce performance and lead to overreaching/overtraining. Moreover, Smith [21] suggested the “cytokine hypothesis” characterized by symptoms including a local acute inflammatory response with release of IL-1beta, IL-6 and TNF-a, evolving into chronic inflammation related to the OTS.

### 2.1. Inflammation and Peripheral Signals in Elite Cyclists

Professional cyclists are a class of highly trained athletes subjected to different schedules of training and often to ultra-endurance training. Serrano et al. [22] reported an anti-inflammatory response after a road cycling competition that is a combination of endurance and resistance series with sprint bouts. This response is related to the increase in an antioxidant and anti-inflammatory stress hormone, melatonin that can modulate the inflammatory state reducing OS. In these athletes, load intensity reached submaximal and maximal strength and power.

Nieman et al. [23] reported in a heterogeneous group of cyclists, and after an intense endurance exercise, a modification in inflammation and innate immunity. The athletes cycled for approximately two hours at 60% watts combined with a 10-km cycling time trial. They found immediately post-exercise an increase in the plasma level of cytokine IL-6, tumor necrosis factor-alpha, granulocyte-macrophage colony-stimulating factor, interferon-g, IL-1b, IL-2, IL-8, IL-10 and IL-12p70. In a recent study performed in trained elite cyclists, the same authors [24], using metabolomics experiments, reported an increase in the oxidized linoleic acid derivative 13-Hydroxyoctadecadienoic acid and 9-Hydroxyoctadecadienoic acid (13-HODE and 9-HODE) after a prolonged cycling trial but this increase was not correlated with increases in plasma cytokines IL-6, IL-8 and IL-10. HODEs are stable lipid peroxidation products that increase in several deseases and little information is present on the relationship between 13-HODE and 9-HODE changes in plasma and oxidative stress and inflammation in athletes, hovewer, the authors reported that following prolonged and intensive exercise, endurance athletes show a modification in the lipid pathway. Data from this study do not relate HODES to inflammatory cytokines within an exercise context but they reported a significant increase immediately post-exercise, suggesting an increase in their oxidation during prolonged exercise and the inclusion of HODEs as a stable oxidative stress biomarker in training [24]. Lakhdar et al. [25] reported the effect of maximal exercise after intense training on plasma adiponectin and leptin. They reported that before training, plasma adiponectin is not modified after maximal exercise, but after six months training, athletes showed an increase in adiponectin concentrations. On the contrary, after six months of training, there is a decrease in leptin concentrations.

They suggest a possible link between the increase in adiponectin and the training improvement due to the endurance or resistance training in cyclists. Córdova Martínez et al. [26] determined the effects of repetitive and intense exercise (a multi-stage cycling race) on several cytokine plasma levels. They observed an accumulation of pro-inflammatory cytokines IL6, TNFa, interferon c (IFNc) and IL2. In summary, the increase in IL6 showed by most of the authors could be due to a local inflammatory response, as it is known that muscle repair includes an initial pro-inflammatory phase and induces the expression of cytokines [27] able to stimulate muscle adaptation. Without a suitable recovery period, this inflammatory state could be responsible for muscle damage due to the increasing production of reactive oxygen species (ROS). In conclusion, cycling competitions represent an important physical overload even for well-trained athletes because they may lead to physical exhaustion. In these conditions, IL-6 increased to activate the expression of several anti-inflammatory cytokines. However, the increase in adiponectin after training sessions can be a sign of improving performance.

### 2.2. Inflammation and Peripheral Signals in Elite Basketball Players

In young well-trained female basketball players, Plinta et al. [28] found, as shown for other sports, a significant decrease in plasma ghrelin and leptin levels after a long-term moderate aerobic exercise, whereas plasma adiponectin remained unchanged. On the contrary, after short-term moderate aerobic exercise, they found no changes in plasma ghrelin and leptin levels but an increase in adiponectin. The study conducted by Schmidt et al. [29] in young not training volunteers and after a treadmill exercise at 50 %, 70 % and 90 % of maximum oxygen consumption shows no change of plasma ghrelin. Chatzinikolaou et al. [30] performed a study after a basketball match and during a six-day simulated in-season microcycle. They demonstrated that a basketball match increases the plasma levels of pro-inflammatory cytokines IL-1ß and IL6 due to muscle microtrauma. Moreover, the impact on muscular damage and inflammatory response was reported by the study of Souglis et al. [31], in which inflammatory responses and muscle damage in different types of sports were compared. Male elite players of four different sports: soccer, basketball, volleyball and handball, were analyzed. Inflammation (indicated by TNFa, IL6 and C-reactive), muscle damage (represented by increase in creatine kinase CK and lactate dehydrogenase LDH activity) and metabolic stress (urea and ammonia production) show an intermediate stress for basketball players. The authors suggested that the increase in ROS promotes ILs production and inflammation. In summary, in basketball players, an increase in IL6 is due to muscle damage that leads to local inflammation.

### 2.3. Inflammation and Peripheral Signals in Elite Soccer Players

The study of Souglis et al. [32] reported above, in which inflammation and muscle damage in different sports including soccer were compared, shows an increase (3–4-fold) in inflammatory cytokines (among them IL-6) and muscle damage for this sport.

Unal et al. [33] carried out a study on the chronic effect of training (training for 1.5 h a day, one match and one day rest) in professional soccer athletes and they found a reduction in resting leptin levels and a negative correlation between serum leptin and maximal consumption of oxygen (VO2Max). In conclusion, regular and long-term exercise decreases leptin levels and increases fat metabolism. Romagnoli et al. [34] reported an increase in CK and IL6 in twenty young male professional soccer players that played a 90-min soccer match. The authors concluded that a soccer match induces muscle damage that leads to an inflammatory state.

Martín-Sánchez et al. [35], using a proteomic approach, demonstrated that elite soccer players submitted to an intensive training showed differences in the content of plasma proteins associated with inflammatory/oxidative stress in comparison to recreational soccer players. The authors reported that professional soccer players showed in particular an increase in an anti-inflammatory factor, alpha-1 antichymotrypsin. They proposed that regular exercise reduces inflammation, hovewer, they did not find changes in pro-inflammatory biomarkers (IL-6). In conclusion, they suggest that regular training stimulates an anti-inflammatory state without modifications in pro-inflammatory molecules. It is interesting to point out, hovewer, that Alpha-1 antichymotrypsin levels are associated with the control of oxidative damage and throughout a 72-h recovery period, a soccer match increases the levels of oxidative stress [36].

In conclusion, in elite soccer players, more than in other athletes, an intensive and inadequate training program modifies the inflammatory status, and this may be associated with a reduction in performance.

### 2.4. Inflammation and Peripheral Signals in Elite Swimmers

Karamouzis et al. [37] reported in swimmers after intense prolonged exercise (25 km swim race in 6.9–10.5 h) a reduction in plasma leptin due to the negative energy balance. As reported, leptin reduced the appetite by reducing the level of a potent, long-acting vasoconstrictor peptide, the neuropeptide Y (NPY) in the hypothalamus. Appropriate changes of leptin and NPY take place in order to compensate the negative energy balance established in swimmers due to prolonged effort. Knab et al. reported that in elite sprint and middle-distance swimmers, swim workouts (high-intensity intervals 1:1, 1:2 swim-to-rest ratio) induced little inflammation, high levels of oxidative stress and immune changes [38]. José Mário Morgado et al. reported that elite swimmers during a heavy training period showed an increase in production of inflammatory mediators [39]. In conclusion, swimmers showed a chronic elevation in oxidative stress in all the studies analyzed and this supports the idea that long-term intensive training may affect the function of immune cells.

## 3. Redox Homeostasis and Oxidative Stress in Sports

Aerobic and anaerobic exercises result in alterations of redox homeostasis in untrained, trained and well-trained athletes. Following intensive physical activity in elite athletes, the source of systemic oxidative stress (OS) is not fully understood but it is proposed that skeletal muscle is the main contributor to the exercise-induced ROS [40]. In this tissue and during muscle contraction, the loss of mitochondrial electrons contributes only marginally to the production of ROS whose primary sources are NADPH oxidase, xanthine oxidase, nitric oxide (NO) synthase and release of arachidonic acid from cell membranes [40]. Moreover, several different events such as lactate accumulation, catecholamine oxidation, body temperature increase, post-exercise inflammation, cytokine secretion and respiratory burst contribute to increase OS. The ROS produced in skeletal muscle diffuses out of the cell and plays an important signaling role in several physiological and metabolic events such as muscle contraction, cell growth, proliferation and immune function, and moreover, they exhibit physiological stimulus for muscle regeneration. ROS upregulate the expression of antioxidant enzymes leading to a stimulation of the antioxidant defense system. However, an increase in ROS production or a reduction in antioxidant capacity results in an imbalance and the redox state becoming pro-oxidant causes molecular damage [41]. Elite athletes have a very strong antioxidant defense system but a prolonged and strenuous exercise without an adequate rest period can lead to oxidative damage producing protein oxidation and developing muscle fatigue, injury and overreaching/overtraining with the impairment of sport performance. There is a fine line between the oxidative stress/inflammation induced by exercise that increases performance and the oxidative stress due to excessive effort that causes fatigue and muscle damage. It seems that there is a U-shaped association between ROS/inflammation and performance: if ROS/inflammation increases, so does the performance, but only up to a limit, after which, as ROS/inflammation continues to increase, the performance decreases [42]. Cazzola et al. [43] found an increase in lipoperoxide levels and erythrocytes membrane fluidity lipoprotein in a group of professional soccer players. Moreover, they reported an increase in plasmatic antioxidants (ascorbic and uric acid, α -tocopherol) and superoxide dismutase (SOD) activity. In a pilot study, Al-Khelaif et al. [44] identified the metabolic signature that differentiates elite athletes practicing different sport in order to understand their adaptation to training. It may be useful to plan future training programs and improve performance. The authors found changes in sex steroid hormones biosynthesis and OS substrates (glutathione metabolism).

The influence of seasons on training has been analyzed by Balog et al. [45] measuring several parameters regarding lipoprotein oxidation (LPO) and antioxidant enzymes plasma levels; they reported a beneficial effect of long-term exercise in men during autumn/winter and, on the contrary, in women these positive effects are reported during summer. Michalickova et al. [46] in a longitudinal study determined that in winter, an intense training period in elite athletes reduced the antioxidant defense and increased the effect on lipid and protein oxidation. The authors suggested that these results might increase susceptibility to muscle damage. Most of the studies on OS markers and antioxidant levels are conducted in male athletes but since men and women show different hormonal statuses and different ratios of progesterone/estradiol, differences in redox status between them could be found. Arsic et al. [47] demonstrated that prolonged strenuous physical activity increases lysophosphatidylethanolamine (LPE), a natural byproduct of lipid peroxidation, total antioxidant status, H_2_O_2_, glutathione disulfide (GSSG) level and catalase (CAT) activity in well-trained female athletes who practice anaerobic/aerobic sports. They conclude that these results could be related to the adaptation mechanisms of antioxidative defense that depends on the type of sport. The results of a study by Souglis et al. [31] showed that following a soccer match, sex and playing position show different effects on OS and muscle damage. The authors reported that for the same player position, there are higher OS values in males compared with females. They suggested that female muscles have a lower load work during soccer games and estrogens play a protective role in the process of exercise-induced muscle damage. In a recent paper [48], we suggested that a specific post-activation potentiation training protocol (PAP) is able to increase force and performance in different sports activities, without increasing the level of OS. Recently, for the first time, the impact of different natural altitude training strategies (training in hypoxia) on the redox status of elite athletes has been analyzed. León-López et al. [49] reported the impact of different altitudes (moderate, low and near-sea level) on the redox status of elite swimmers. Their results showed that high altitudes induce a significant alteration in redox homeostasis compared with the sea level controls.

Altitude exposure can increase markers of OS after acute exercise and this is observed in endurance-trained cyclists. McGinnis et al. [50] simulated altitude in an environmental chamber and they found an increase in LPE after cycling at 60% at an altitude of 3000 m in comparison to 975 m of altitude. Recently, Wadley et al. [51] evaluated the independent effect on OS of acute exercise and hypoxic exposure comparing two different training habits: sea level and 2000 m above sea level. At the same exercise intensity and immediately post-exercise, at high level, there is an increase in plasma CA activity and total protein carbonylation (PC) content in comparison to sea level and the authors reported a decrease in PC when the exercise was performed in normoxia. Several authors reported that exercise training led to the production of ROS causing an increase in PC. In a previous paper [52], we reported that plasma PC depends on the degree of OS in the compartment where the protein originated. In endurance athletes, we found (a) several plasma proteins that undergo carbonylation in response to training, (b) proteins whose carbonylation was not affected and (c) proteins that were carbonylated in resting conditions.

Different studies demonstrated damage associated with OS in DNA following exhaustive/high-intensity endurance exercise, eccentric exercise and unaccustomed exercise. However, this effect is recovered after adequate rest. DNA damage is measured with an increase in 8-hydroxy-2′-deoxyguanosine (8-OHdG) plasma levels because the activity of several DNA-repairing enzymes (among them: human 8-oxoguanine DNA glycosylase1 and oxidized purine-nucleoside triphosphate) is increased to protect against exercise-induced DNA damage. Yasuda et al. [53] determined the effects of two races of endurance exercise on oxidative DNA damage in trained cyclists. No cumulative effects at the muscle or urinary levels of 8-OHdG between two repeated sessions were found, probably due to the adaptive response dependent on the athlete’s training state.

### 3.1. Redox Homeostasis and Oxidative Stress in Elite Cyclists

In a short report, Lekhi et al. [54] analyzed the influence of strenuous exercise on the redox system of trained elite cyclists after exhaustive endurance training. The authors reported an increase in the plasma levels of serum malondialdehyde (MDA), uric acid, vitamin E and vitamin C. They found an increase in SOD activity and a low CAT activity, suggesting an inactivation of the defense system due to an excessive production of free radicals. They showed an increase in LPE suggesting that endurance training in cyclists modifies the activities of the two erythrocyte scavenger enzymes SOD and CAT but this is not sufficient to counteract the increase in ROS and could lead to an increase in LPE. In order to understand if consecutive days of high-intensity exercise can induce OS in highly trained cyclists, Shing et al. [55] examined the influence of strenuous exercise (they reported three consecutive days of high-intensity cycling) on OS levels in blood and urine. The results were an initial increase in plasma MDA, total antioxidant status (TAS) and urinary allantoin excretion. After strenuous exercise, the TAS concentration in plasma was elevated. No significant changes in urine were reported. Muñoz et al. [56] suggested that strenuous exercise (they reported periods of a short time of high-intensity cycling) leads to an increase in LPE and MDA plasma levels and a reduction in vitamin C in erythrocytes. On the contrary, no statistical changes were observed in sub-maximal-intensity exercise. Regarding how different types of training could influence OS in highly trained cyclists, Leonardo-Mendonça et al. [57] investigated the effect of five different phases of training: 1—a control period of seven days of rest; 2—a hard training of six days; 3—a progressive training of four days; 4—one-day competition; and 5—a 7-day recovery period. They supplemented two groups of athletes with vitamins C and E, respectively, and a third group was the control. To measure the antioxidant effects on plasma redox status, they used the LPO/ORAC−1 ratio (oxygen radical absorbance capacity—ORAC). They reported that in phase 1, supplementation with vitamins C and E reduces lipoperoxidation (LPO) but it does not prevent LPO increase after intensive training periods. The control group without vitamins supplementation improved significantly the LPO/ORAC−1 ratio after phase 3. They showed that there is no correlation between changes in the erythrocyte oxidized/reduced glutathione ratio (GSSG/GSH−1) and the antioxidant vitamin intake. They conclude that well-trained athletes do not need further antioxidant supply and periods of appropriate training cause beneficial adaptation of the antioxidant defenses. The intake of vitamins could delay the adaptation and negatively interfere with training efficiency. Recently Córdova Martínez et al. [26] reported that in well-trained cyclists, the recovery period between stages is enough to normalize the redox state. They reported a reduction in GSH and an increase in GSSG levels in erythrocytes and they suggested that muscle capitation of GSH could explain its decreases in plasma and as a conclusion, they proposed a mechanism to protect skeletal muscle from oxidative damage during physical activity. They observed an increase in glutathione reductase (GR) activity that could be a response to recover GSH after the increase in GSSG. Regarding LPE, they found an increase in F2-isoprostanes due to the increase in oxygen radicals considering that F2-isoprostanes are produced by the oxidation of phospholipids.

In a recent study, Maleki et al. [58] reported changes in OS markers on seminal plasma after long-term high-intensity training in elite cyclists. The authors reported an increase in ROS and MDA levels and in SOD and CA activity; moreover, they measured various free radical damage products as total antioxidant capacity (TAC) levels that resulted as reduced. They reported that several conditions during intense exercise, such as increased oxygen uptake (VO2max), local tissue hypoxia or elevated tissue temperatures, could contribute to increase OS in seminal plasma. They also reported that an increase in temperature to the testicles inhibits testosterone secretion and could contribute to induce an imbalance in the oxidant/antioxidant ratio after long-term high-intensity training. In conclusion, cyclists that follow an ultra-endurance training could adapt themselves to ROS production and as a consequence prevent the OTS that follows high-intensity training.

### 3.2. Redox Homeostasis and Oxidative Stress in Elite Basketball Players

During the season, elite basketball players train twice a day and play one or two games per week. Even though both aerobic and anaerobic systems are activated, several studies have demonstrated that anaerobic metabolism is the primary energy pathway activated in basketball players because basketball requires movements with variable velocity, continuous accelerations, decelerations, stopping and sudden changes of direction. These authors reported that elite basketball players show good anaerobic power [59] and moderate aerobic capacity [60] when compared to other team sport athletes [61].

The overall duration of a typical basketball match is 40–48 min, in which an athlete carries out a combination of high-intensity actions (sprinting, running, jumping, shuffling) interspersed with low-intensity activities (jogging, walking) and active or passive recovery [62]. In one study, Spanidis et al. [63] found that at the end of the season, players have a more severe OS than at the beginning of it due to the high number of matches played. Authors found at the end of the season a high level of the static oxidation–reduction potential marker (sORP) and TAC, and a lower level of GSH, suggesting that the balance between the oxidant and reductant is in favor of the oxidant. Moreover, the OS at the end of the season was not so severe, probably due to the increases in the antioxidant defense mechanisms. Similar data were obtained in another study that considered the OS, total antioxidant status and vitamin levels in adolescent professional basketball players [64]. Researchers found that young trained players have an increase in plasma TAC. In addition, athletes have elevated values of the antioxidant vitamins A, E and B6 in plasma, indicating a redox systems adaptation to the training that may compensate the OS. Hadžović and colleagues [65] investigated the impact of training on the oxidation status of professional wrestlers, soccer players and basketball players. OS markers increased in all types of sports without differences, suggesting that training leads to increased ROS levels, followed by increased antioxidant capacity. The study of Perrea and colleagues [66] found that 15 min from the end of the game, basketball players show an increase in total peroxides in serum but a reduction in polymorphonuclear elastase (an oxidative and inflammatory marker) compared to soccer players. However, several reports using serum peroxide as a marker for the OS show ambiguous results, probably because the time in which the blood sample is taken is crucial for this analysis [67]. We can conclude that a basketball match elicits moderate inflammatory responses associated with intense eccentric muscle activity that induces muscle microtrauma and an increase in ROS production. Moreover, basketball players show a redox systems adaptation to the training that may compensate the OS.

### 3.3. Redox Homeostasis and Oxidative Stress in Elite Soccer Players

The physical demands activity required for soccer players includes: sprints, change of direction and high-intensity running involving both aerobic and anaerobic energy pathways leading to alterations of biological processes such as inflammation, muscle damage and OS. Jakovljević et al. [68] investigated the effects of a progressive maximal physical effort test on peripheral blood molecules in elite soccer players. The authors reported an initial increase in NO2- production for an increased blood flow to skeletal muscle, and this value declines during physical effort due to inactivation by ROS, thus suggesting a role of free radicals as signals to stimulate the adaptive processes. Fatouros et al., 2010 [69], suggested that a single competitive event induces time-dependent modifications in OS. They reported an increase in the marker of muscle damage (delayed onset of muscle soreness DOMS) and CK as well as an increase in markers of inflammation (leucocytosis). The authors suggested that leukocytes generate ROS that promote post-exercise inflammation and oxidize lipids and proteins. Moreover, as reported by others, they found an oxidation of plasma protein. For the first time, they found an elevated protein oxidation level measured as total protein carbonylation (PC): it could be the result of increased oxidation of albumin and other serum proteins. The ratio GSH/GSSG declined during the recovery period and at the same time, an increase in ammonia, uric acid and hypoxanthine levels was found, indicating a transient OS. When neutralizing the ROS formed in the muscle cell, the oxidation of GSH to GSSG exceeds its reduction by enzymes, and the GSSG is exported to maintain a constant GSH/GSSG ratio in the plasma. Therefore, the increase in the concentration of GSSG and the consequent reduction in the plasma GSH/GSSG ratio indicate that the production of free radicals has increased. The exercise intensity cause increased GSSG levels and this increase is related to muscular fatigue. It is often observed an increase in GSSG without variation in GSH plasma levels. This is because other organs including the liver provide adequate GSH availability. Therefore, monitoring physiological responses to acute exercise, including oxidative stress and antioxidant measures, could be a useful additional tool in determining the need for adequate recovery in athletes [69]. The studies of Gravina et al. [70,71] confirm that in an elite female team, a single match has a temporary effect on body metabolism. This suggests a sequence of events between cell breakdown and the increase in antioxidant capacity: after the match, the cell breakdown increases the CK level that increases the level of LDH responsible for the increase in uric acid and albumin, and these lead to total antioxidant status. Moreover, they found an increase in serum testosterone levels just after the match, suggesting a possible role of this hormone to increase force in muscle activity involving a short contraction time. Zivkovic et al. [72] determined the effects of a training program on the basal redox status of young male soccer players. They measured the classical OS markers and they found that, after the period of the training program, the levels of thiobarbituric acid reactive substances (TBARS), a byproduct of lipid peroxidation and NO2 were significantly increased, while O^2–^ and H_2_O_2_ remained unchanged. On the other hand, SOD and CAT activity increased, while GSH decreased. Le Moal et al. [73] followed up the variation of pro-/antioxidant status in erythrocytes and plasma throughout a whole season in elite professional soccer players from the French league and correlated these variations with training load. They found modifications in the GSH/GSSG ratio, which evolved during the season periods. Antioxidant enzymes display no significant modifications throughout the season. In conclusion, their results suggest that the redox status of professional soccer players is altered according to training period (in-season periods) and that GSH/GSSH ratio variations are correlated with cumulated training loads.

In summary, oxidative stress is markedly upregulated after a soccer game and it is correlated with cumulated training loads during the football season.

### 3.4. Redox Homeostasis and Oxidative Stress in Elite Swimmers

Competitive swimming combines factors such as the simultaneous contribution of arms and legs to propulsion, water immersion and prone position and allows muscles to work in harmony and in accordance with each other, since gravity drops down to almost zero in water. Unlike other popular sports, the OS that occurs in swimming is largely affected by the type of competitions, and swimmers require a sophisticated training for both aerobic (endurance exercises) and anaerobic (short high-effort exercises below 200 m) exercise to increase performance. Several studies indicate that swimming is involved in OS homeostasis changing but the duration and intensity of the stress are strictly correlated with the effort [74]. The study of Lubkowska and colleagues [75] reports that swimming training in winter makes athletes more able to respond to stressful conditions. They found that these conditions induce an increase in the expression of antioxidant systems (GR, glutathione S-transferase GST, CAT and SOD). Authors suggest that a positive adaptive change occurs in the antioxidant system of winter swimmers that seems to increase the readiness of the human body to stress factors. The study of Deminice et al. [74] investigated the modulations of OS biomarkers induced by high-intensity interval training (100 m maximum swims with 10 min intervals) bouts and their relationship to swimming performance. High-intensity interval training is a standard method to increase the body response to anaerobic exercise. Interestingly, researchers observed that lactate increases especially after the third interval, and subsequently its value tends to stabilize. They suggested that the ability to produce and to tolerate high levels of blood lactate during the effort is associated with a successful performance. Concerning the redox status induced by high-intensity interval training, they noted an increase in formation of TBARS as indices of LPE, and CK due to OS of anaerobic exercise; moreover, they found an increase in GSH and ascorbic acid as markers of the antioxidant defense systems modulation. On the contrary, Kabasakalis et al. [76] investigated the effect of the aerobic effort of ultramarathon swimming (50 km) on blood OS status in well-trained swimmers. The ultramarathon swimming is a useful model to study OS because it combines a long exercise duration with low intensity and dissociates the production of ROS from muscle damage. Interestingly, researchers found that the ultramarathon induces no significant increase in blood oxidative stress markers in well-trained athletes. The authors suggested that there is an intensity training level below which oxidative stress does not occur. Another study of the same authors [77] compared the effect of aerobic and anaerobic efforts on the oxidative status of young trained swimmers. They found that both endurance and high-intensity exercise perturbed the redox balance without inducing prolonged oxidative damage. In fact, the alteration in redox homeostasis is soon recovered to the baseline. Interestingly, 8-hydroxy-2′-deoxyguanosine, a marker of DNA oxidation, increased after the anaerobic trial, suggesting the importance of exercise intensity in ROS homeostasis alterations. Other markers such as MDA, PCs and GSH showed no modification because the antioxidant defense system may limit the potential negative effects of ROS formation during the training.

## 4. Conclusions

The purpose of this review was to focus on high-performance exercise studies performed in athletes to correlate peripheral mediators and key inflammation markers with physiological and pathological conditions in different sports such as basketball, soccer, swimming and cycling. The main source of discrepancies among reviewed studies indeed mainly concerns differences in the type, duration and intensity of training (Table 1). In well-trained athletes, a decrease in plasma concentration of leptin and an increase in adiponectin and ghrelin were observed. The increase in adiponectin plasma concentration was followed by a reduction in pro-inflammatory cytokines (such as IL-1β, IL-6 and TNFα). The adiponectin response could thus be implicated in inflammation attenuation and in the induction of muscle adaptation typical of an effective training. An altered modulation of this peripheral response may conversely be indicative of training stress, prolonged fatigue and possible overreaching. Although it is known that elite athletes have a very strong antioxidant defense system, oxidative damage could induce muscle fatigue, injury and overreaching/overtraining with consequent impairment of sport performance (Table 2). Therefore, the possibility to use redox markers among indicators of proper training is crucial. We suggest that the antioxidative status is the best indicator of proper training. Total plasma protein carbonylation (PC) and lipid oxidation (TBARS and MDA) could be useful markers of a reduction in antioxidant defense after a strenuous and prolonged training period without an adequate rest period. 

## Figures and Tables

**Table 1:** Inflammation marker measured in cycling, basketball, soccer and swimming.

Marker	Basketball				Cycling				Soccer				Swimming			
		Effort *	Effect	Ref.		Effort *	Effect	Ref.		Effort *	Effect	Ref.		Effort *	Effect	Ref.
IL1-β	s	54.5 ± 2.9	increase	[30]	p	4-days competition	not change	[22]					iv **	season	decrease	[39]
					p	73.2 ± 6.7	not change	[26]								
					p	55.8 ± 8.4	increase	[23]								
IL2					p	73.2 ± 6.7	increase	[26]								
					p	55.8 ± 8.4	increase	[23]								
IL6	s	game	increase	[31]	p	4-days competition	increase	[22]	p	53.5±1.2	increase	[35]	iv **	season	decrease	[39]
	s	54.5 ± 2.9	increase	[30]	p	73.2 ± 6.7	increase	[26]	s	game	increase	[31]				
					p	51.7 ± 1.4	increase	[24]								
					p	55.8 ± 8.4	increase	[23]								
IL8					p	51.7 ± 1.4	increase	[24]								
					p	55.8 ± 8.4	increase	[23]								
IL10					p	51.7 ± 1.4	increase	[24]								
					p	55.8 ± 8.4	increase	[23]								
IL12					p	55.8 ± 8.4	not change	[23]					iv **	season	decrease	[39]
TNF-α					p	4-days competition	increase	[22]	s	game	increase	[31]	iv **	season	decrease	[39]
	s	game	increase	[31]	p	73.2 ± 6.7	increase	[26]								
					p	51.7 ± 1.4	increase	[24]								
					p	55.8 ± 8.4	increase	[23]								
INF-γ					p	73.2 ± 6.7	increase	[26]								
					p	55.8 ± 8.4	increase	[23]								
GM-CSF					p	51.7 ± 1.4	increase	[24]								
					p	55.8 ± 8.4	increase	[23]								
MCP-1					p	51.7 ± 1.4	increase	[24]								
Adn	p	3-months training	not change	[28]	p	72.1 ± 8.8	increase	[25]								
Leptin	p	3-months training	not change	[28]	p	72.1 ± 8.8	decrease	[25]	s	63.7 ± 5.5	decrease	[33]	s	intense exercise	decrease	[37]
													s	cold water swimming	decrease	[38]
Ghrelin	p	3-months training	decrease	[28]												
Visfatin	p	3-months training	decrease	[28]												
C-Reactive Protein	s	54.5 ± 2.9	increase	[30]					s	game	increase	[31]				
	s	game	increase	[31]												

* Sport effort is described by the VO2max value (mL/Kg/min) which refers to the maximum amount of oxygen consumption. Where not reported, effort is defined by brief description of the analysed exercise. Tumor necrosis factor (TNF), interferon (IFN), granulocyte-macrophage colony-stimulating factor (GM-CSF), monocyte chemoattractant protein-1 (MCP-1), Adiponectin (Adn). Biological samples: p = plasma; s = serum, ** iv = in vitro analysis of monocytes stimulated with LPS.

**Table 2:** Oxidative stress marker measured in cycling, basketball, soccer and swimming.

Marker	Basketball			Cycling			Soccer			Swimming		
		Effort *	Ref.		Effort *	Ref.		Effort *	Ref.		Effort *	Ref.
CAT	s	54.5 ± 2.9	[30]	p	63.7 ± 5.3	[51]	s	59.7 ± 3.1	[69]	s	winter-swimming	[75]
	e	season	[63]	s	Exhaustive exercise	[54]	s	53.9	[72]			
				e	73.2 ± 6.7	[26]						
				sp	63.8 ± 5.2	[58]						
SOD				s	Exhaustive exercise	[54]	p	game	[71]	s	winter-swimming	[75]
				s	73.2 ± 6.7	[26]	s	53.9	[72]			
				e	76.0 ± 4.0	[55]	e	season	[73]			
				sp	63.8 ± 5.2	[58]						
TAC	p	training	[64]	p	76.0 ± 4.0	[55]	p	game	[71]	p	ultramarathon	[76]
	p	season	[63]	p	hard training	[57]	p	training	[65]	s	winter-swimming	[75]
	p	training	[65]	p	51.7 ± 10.5	[50]	s	59.7 ± 3.1	[69]			
	s	54.5 ± 2.9	[30]	sp	63.8 ± 5.2	[58]						
GPX	wb	54.5 ± 2.9	[30]	e	76.0 ± 4.0	[55]	p	game	[71]	s	winter-swimming	[75]
				e	4-days competition	[22]	e	season	[73]			
				e	73.2 ± 6.7	[26]	wb	59.7 ± 3.1	[69]			
GR				e	4-days competition	[22]	p	game	[71]	s	winter-swimming	[75]
				e	73.2 ± 6.7	[26]						
GST										s	winter-swimming	[75]
MPX	s	training	[66]				s	training	[66]			
LDH							p	game	[71]			
PCs	p	season	[63]	p	51.7 ± 10.5	[50]	wb	59.7 ± 3.1	[69]	p	ultramarathon	[76]
	p	54.5 ± 2.9	[30]	p	63.7 ± 5.3	[51]				p	different exercise setting	[77]
GSH	e	season	[63]	p	73.2 ± 6.7	[26]	p	53.9	[72]	p	high intensity bouts	[74]
	e	54.5 ± 2.9	[30]	e	4-days competition	[22]	wb	59.7 ± 3.1	[69]	s	winter-swimming	[75]
				e	hard training	[57]	wb	season	[73]	e	different exercise setting	[77]
				e	73.2 ± 6.7	[26]						
GSSG	e	54.5 ± 2.9	[30]	p+e	73.2 ± 6.7	[26]	wb	59.7 ± 3.1	[69]	s	winter-swimming	[75]
				e	4-days competition	[22]	wb	season	[73]			
				e	hard training	[57]						
Lactate				p	65.5 ± 9.3	[56]	p	59.7 ± 3.1	[69]	p	different exercise setting	[77]
										wb	high intensity bouts	[74]
Bilirubin							p	game	[71]	p	different exercise setting	[77]
Oxigen radicals	p	training	[64]	p	73.2 ± 6.7	[26]	p	59.7 ± 4.6	[68]			
	s	training	[66]	sp	63.8 ± 5.2	[58]	p	53.9	[72]			
							s	training	[66]			
Uric acid				s	Exhaustive exercise	[54]	p	game	[71]	p	different exercise setting	[77]
							wb	59.7 ± 3.1	[69]			
Vitamin A	s	training	[64]				pl	season	[73]			
Vitamin B6	s	training	[64]									
Vitamin E	s	training	[64]	p	76.0 ± 4.0	[55]	p	season	[73]	p	high intensity bouts	[74]
	s	training	[64]	p	65.5 ± 9.3	[56]						
				p	hard training	[57]						
Vitamin C				p	65.5 ± 9.3	[56]				p	high intensity bouts	[74]
				p	hard training	[57]						
				s	Exhaustive exercise	[54]						
8-OHdG				p	hard training	[57]				p	different exercise setting	[77]
				u+m	60.0 ± 5.1	[53]						
8-Isoprostane				u	73.2 ± 6.7	[26]				p	winter-swimming	[75]
MDA	p	training	[65]	p	76.0 ± 4.0	[55]	p	training	[65]	p	different exercise setting	[77]
	p	54.5 ± 2.9	[30]	p	65.5 ± 9.3	[56]	wb	59.7 ± 3.1	[69]			
				p	4-days competition	[22]						
				p	hard training	[57]						
				p+e	73.2 ± 6.7	[26]						
				s	Exhaustive exercise	[54]						
				sp	63.8 ± 5.2	[58]						
TBARS	p	season	[63]	p	63.7 ± 5.3	[51]	p	53.9	[72]	p	high intensity bouts	[74]
										p	ultramarathon	[76]
AOPP	p	season	[63]				p	game	[71]	p	high intensity bouts	[74]
	p	training	[65]				p	training	[65]			
ORAC				p	hard training	[57]						
FRAP				p	51.7 ± 10.5	[50]						
				p	63.7 ± 5.3	[51]						
sORP	p	season	[63]									
TOS										s	winter-swimming	[75]
Allantoin				u	76.0 ± 4.0	[55]						

* Sport effort is described by the VO2max value (mL/Kg/min) which refers to the maximum amount of oxygen consumption. Where not reported, effort is defined by brief description of the analysed exercise. Superoxide dismutase (SOD), catalase (CAT), total antioxidant capacity (TAC), glutathione peroxidase (GPX), glutathione reductase (GR), lactate dehydrogenase (LDH), glutathione S-transferase (GST), Myeloperoxidase (MPX), protein carbonyls (PCs), Glutathione (GSH), oxidized glutathione (GSSG), 8-hydroxy-2’-deoxyguanosine (8-OHdG), malondialdehyde (MDA), thiobarbituric acid reactive substances (TBARS), advanced oxidation protein products (AOPP), Oxygen radical absorbance capacity (ORAC), ferric reducing antioxidant power (FRAP), (A) static oxidation reduction potential (sORP), total oxidant status (TOS). Biological samples: e = erythrocytes; m = muscle; p = plasma; sp = seminal plasma; s = serum; u = urine; wb = whole blood.
